# *HOXA9* gene promotor methylation and copy number variation of *SOX2* and *HV2 genes* in cell free DNA: A potential diagnostic panel for non-small cell lung cancer

**DOI:** 10.1186/s12885-023-10793-7

**Published:** 2023-04-10

**Authors:** Abla Abou-Zeid, Doaa Hashad, Ayman Baess, Mai Mosaad, Eman Tayae

**Affiliations:** 1 Department of Clinical and Chemical Pathology, Faculty of Medicine, Alexandria University, Alexandria, Egypt; 2 Department of Chest Diseases, Faculty of Medicine, Alexandria University, Alexandria, Egypt

**Keywords:** Lung cancer, Non-invasive, *HOXA9*, *SOX2*, *HV2*, Methylation

## Abstract

**Background:**

Most cases of lung cancer are diagnosed at advanced stage. Detection of genetic and epigenetic markers in cell-free DNA (cfDNA) is a promising tool for the diagnosis of lung cancer at an early stage. The aim of this study was to identify non-invasive diagnostic markers in cell free DNA (cfDNA) for non-small cell lung cancer (NSCLC) as it is the most common type of lung cancer.

**Methods:**

We investigated the cfDNA *HOXA9* gene promotor methylation by pyrosequencing. Copy number variation of *SOX2* and *HV2* genes were detected by real-time PCR in cfDNA extracted from plasma samples of 25 newly diagnosed NSCLC patients and 25 age and sex matched controls.

**Results:**

Methylation level of *HOXA9* was significantly higher in NSCLC patients than controls (*p* > 0.001). *SOX2* showed significantly higher CNV and *HV2* showed lower CNV in patients than controls (*p* > 0.001, p = 0.001 respectively). Receiver Operating Characteristic (ROC) curve analysis for *HOXA9* methylation, *SOX2* CNV and *HV2* CNV showed a discrimination power of 79.4%, 80% and 77.5% respectively and the area under the curve for the combined analysis of the three genes was 0.958 with 88% sensitivity and 100% specificity.

**Conclusions:**

In this study, we suggest a potentially diagnostic panel that may help in detection of lung cancer with high sensitivity and specificity using cell free DNA. This Panel included *HOXA9* gene methylation and the CNV of *SOX2* and *HV2* genes.

## Introduction

Lung cancer is the second most common cancer among men and women Worldwide. It represents 13% of all cancer new cases. It is also considered the first cause of cancer related deaths, being responsible for 25% of all cancer deaths [1]. Lung cancer is categorized into two main types according to their origins; small cell (SCLC) and non-small cell lung cancer (NSCLC). NSCLC is the most common type accounting for 80–85% of all lung cancer cases [2]. Unfortunately, most cases are diagnosed in advanced stage due to the non-specificity of early symptoms and the high false positive rate and high cost of low dose computed tomography (CT) preventing its use in population based screening for lung cancer [3, 4]. Therefore, there is a crucial need for non-invasive biomarkers for early diagnosis and risk stratification of lung cancer.

Several studies were done to assess the use of genetic and epigenetic markers in the diagnosis and risk assessment of lung cancer. However, the sensitivity of single molecular marker detection is low, which fails to achieve the standards for diagnosis of lung cancer in an early stage [5–7]. Hypermethylation of gene promoters enriched with CpG islands is one of the epigenetic mechanisms involved in carcinogenesis and was suggested as a potential biomarker for lung cancer. One of the most implicated genes in lung cancer through this mechanism is Homeobox A9 gene (*HOXA9*). *HOX* genes are a large group of genes that act as key regulators of cellular differentiation by encoding the homeodomain proteins. These proteins act as transcription factors controlling genes responsible for cell proliferation, differentiation and cell adhesion [8]. Several studies showed that *HOXA9* promoter hypermethylation leads to its transcriptional inactivation in many types of cancer including lung [9], breast [10], cervix [11], and bladder cancer [12]. Its low expression is associated with epithelial-to-mesenchymal transition (EMT) and may be characteristic of tumor aggression in NSCLC patients [13].

Copy number variation (CNV) is a type of structural variation, in which there is an increase or decrease in the number of copies of a certain gene with subsequent high or low expression of the corresponding protein or noncoding RNA [14]. CNV can affect several signaling pathways as those controlling cell proliferation and apoptosis as sex determining region Y-box 2 (*SOX2*) gene or oxidative phosphorylation as hypervariable region 2 gene (*HV2*) [15]. Dysregulation of *SOX2* expression has been linked to cancer pathogenesis through its role in induction of cellular proliferation mediated by epidermal growth factor receptor (*EGFR)* activation, epithelial-to-mesenchymal transition (EMT), and resistance to apoptosis mediated by B-cell lymphoma 2 like1 (BCL2L1) induced survival signaling [16].

Mitochondrial DNA (mtDNA) is particularly susceptible to damage due to lack of protective introns and histones, and less efficient DNA repair mechanisms leading to sequence mutations or copy number alterations. Most of them occur in the displacement D-loop containing hypervariable region genes *HV1* and *HV2* [17]. Altered mtDNA copy number causes abnormal mitochondrial functions as energy production, signal transduction, apoptosis and cell growth leading eventually to malignant transformation [18, 19].

In this context, our study aimed to evaluate the diagnostic performance of a panel of three genetic and epigenetic markers; *HOXA9* gene promoter methylation, *SOX2* and *HV2* genes copy number variation in circulating cell free DNA in non-small cell lung cancer.

## Patients and methods

The present study was conducted on 50 Egyptian participants divided into 2 groups. The first group included 25 newly diagnosed patients with NSCLC confirmed by histopathological examination admitted to Chest Diseases Department at Alexandria Main University Hospital (Alexandria, Egypt). Patients underwent tumor surgical resection or received chemotherapy or radiotherapy were excluded from the study. The second group included 25 age and sex matched healthy controls.

Blood samples were collected from all subjects after approval of the Committee of Medical Ethics, Faculty of Medicine, Alexandria University.

### Blood sample

Two mL of blood were collected in ethylenediaminetetraacetic acid (EDTA)-containing tubes. Blood samples were mixed thoroughly and plasma was isolated within 2 h from sample collection by centrifugation at 1200 g for 10 min at 4 °C. Plasma was transferred to new micro-centrifuge tubes and centrifuged at 12,000 g for 10 min at 4 °C. Then plasma was transferred to 2-mL tubes and stored at − 80 °C until DNA extraction.

### DNA extraction

Cell free DNA (cfDNA) was extracted from plasma samples using QIAamp DSP Virus Spin Kit (Qiagen, Germany) according to the manufacturer’s recommendations. Concentration and Purity of DNA were assessed using Nanodrop 2000/2000c (Thermo Fisher Scientific, Waltham, MA, USA). Extracted DNA was stored at − 80 °C until further use.

### Analysis of *HOXA9* gene promoter methylation by pyrosequencing

#### DNA bisulphite conversion

A total of 500 ng of plasma cell free DNA was bisulphite converted using EpiTect Fast Bisulfite Kit (Qiagen, Germany) according to the manufacturer’s recommendations.

#### PCR amplification of converted DNA

The PCR reaction was carried out using the Pyromark PCR Kit (Qiagen, Germany). The amplification reaction consisted of 12.5 μl PyroMark PCR Master Mix, 2.5 μl Coral Load Concentrate, 5.5 μl RNase-free water, 2.5 μl of amplification primers with 2 μl of bisulfite-modified DNA (20 ng). *HOXA9* Pyromark CpG assay (hs_hoxa9.05 cpm) included both PCR amplification and sequencing primers. The Thermal cycling was done using SimpliAmp thermal cycler (Thermo Fisher Scientific, Waltham, MA, USA) and included 95 ºC for 15 min, followed by 45 cycles each of denaturation at 94 ºC for 30 s, annealing at 52 ºC for 30 s, and extension at 72 ºC for 30 s and lastly final extension at 72 ºC for 10 min.

#### Methylation analysis by pyrosequencing

**Fig. 1:**
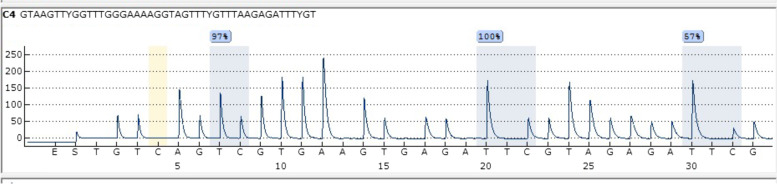
Pyrogram of NSCLC case showing analysis of 3 CpG islands of *HOXA9* promotor methylation by pyrosequencing on pyromark Q24

Pyrosequencing was carried out on 10 μl of the amplified PCR product using sequencing primer and pyroMark gold Kit (Qiagen, Germany) on PyroMark Q24 (Qiagen, USA). PyroMark annealing buffer (Qiagen, Germany,), PyroMark binding buffer (Qiagen, Germany), PyroMark denaturation solution (Qiagen, Germany) and PyroMark wash buffer (Qiagen, Germany) were used. The pyrosequencing analysis was designed to analyze the methylation status of 3 CpG islands (Fig. 1).

### Copy number assays by real-time PCR

#### *SOX2* gene CNV

To assess the *SOX2* gene copy number, TaqMan Copy Number Assays (assay ID: Hs02753059_cn *SOX2* gene (Qiagen, Germany, Cat, no. 4400291), TaqMan Genotyping PCR Master Mix (Applied Biosystems, USA) were used with 20 ng of genomic DNA in a final volume of 20 μL /reaction.

#### *HV2* genes CNV

To measure the *HV2* gene copy number, Maxima SYBR Green/ROX qPCR Master Mix (Thermo Scientific, Rockford, IL, USA) and custom-made primers (10 pmol/reaction) were used with 20 ng DNA/ reaction in a total volume of 20 μL according to manufacturer’s instructions. The *HV2* forward primer sequence was GGGAGCTCTCCATGCATTTGGTA and the *HV2* reverse primer sequence was AAATAATAGGATGAGGCAGGAATC.

Thermal cycling for both *SOX2* and *HV2* gene copy number was performed on Stratagene MX3000P PCR System and included activation of Taq polymerase at 95 °C for 10 min followed by 40 cycles at 95 °C for 15 s for denaturation and 60 °C for 60 s for annealing and extension.

#### Data analysis

The copy number of the target gene was calculated to be two times the relative quantity (RQ). RQ was determined using the comparative Ct (2^−ΔΔCt^) method. *GAPDH* gene was used as reference gene.

### Statistical analysis of the data

Data were analyzed using IBM SPSS software package version 20.0*.* (Armonk, NY: IBM Corp). Qualitative data were described using number and percent. Chi-square test was used to compare between different groups with categorical variables. Fisher's Exact was used for correction for chi-square when more than 20% of the cells had expected count less than 5. The Shapiro test was used to verify the normality of distribution. Quantitative data were described using range (minimum and maximum), mean, standard deviation, median and interquartile range (IQR). Significance of the obtained results was judged at the 5% level. Student t-test was used for normally distributed quantitative variables, to compare between two studied groups, Mann Whitney test was used for abnormally distributed quantitative variables, to compare between two studied groups. Spearman coefficient was used to study the correlation between two abnormally distributed quantitative variables. Receiver operating characteristic (ROC) curve was performed to assess the diagnostic performance of each biomarker and to allow also a comparison of performance between two tests. To evaluate the diagnostic performance of the 3 biomarkers together, logistic regression model with all 3 biomarkers in it was constructed. A ROC curve analysis was then performed based on the probabilties obtained from the logistic regression model. ROC curve and UAC was used to assess the feasibility of using the 3 biomarkers together as a diagnostic tool in discriminating patients with NCCLC from controls.

The sample size calculation was performed using G*power 3.1.9.2 (Kiel, Germany). Based on the following considerations: two tailed, 80% power of the study and 95% confidence level. Power of the study was calculated using Open-Epi software (version 3.01). Power of study was estimated to be 95% made on assumption that percentage of lung cancer patients with elevated % of *HOXA9* methylation above the cutoff value (5%) was 60% (15 out of 25 patients) while the percentage of elevated *HOXA9* methylation above the cutoff value among controls was 12% (3 among 25 controls). The estimated power of study is made at assumption of 95% confidence level.

## Results

### The characteristics of study participants

The NSCLC patients consisted of 23 males (92%) and 2 females (8%). No statistically significant difference was found between cases and controls regarding age and gender. Based on TNM staging, the most prevalent stage among our patients was stage III (40%) and stage IV (40%). Distant metastasis was present in 10 patients (40%). Histopathological examination of the tumors revealed adenocarcinoma in 56% of cases. Clinicopathological data of the studied participant are illustrated in Table 1.

**Table 1:** Clinicopathological data of the studied participant

Characteristics	NSCLC Cases	Controls	Test of Sig	*P*
**Age (years)**				
**Min.–Max**	30.0 – 75.0	34.0 – 78.0	t = 1.671	0.101
**Mean ± SD**	59.04 ± 11.7	53.40 ± 12.16		
**Sex**				
Male	23 (92%)	23 (92%)	χ^2^ = 0.000	^FE^*p* = 1.000
Female	2 (8%)	2 (8%)		
**Smoking**				
Non-smoker	2 (8%)	7 (28%)	**χ**^**2**^ = 3.388	^**FE**^***p*** = 0.138
Smoker	23 (92%)	18 (72%)		
**Pathology**				
SCC	11 (44%)			
Adenocarcinoma	14 (56%)			
**Clinical stage at diagnosis:**				
I	2 (8%)			
II	3 (12%)			
III	10 (40%)			
IV	10 (40%)			

*IQR* Inter quartile range, *SD* Standard deviation, *t* Student t-test, *χ*^*2*^ Chi square test, *FE* Fisher Exact, *pp* value for comparing between the studied groups

### *HOXA9* gene promoter methylation

*HOXA9* gene promoter methylation was significantly higher in patients than controls. Hypermethylation was detected in 60% of cases (15 out of 25 patients). ROC curve analysis showed that at a cut off > 5%, *HOXA9* methylation can detect NSCLC with 60% sensitivity and 88% specificity. Area under the curve (AUC) was 7.94.

### *SOX2* gene copy number variation

Increased *SOX2* copy number was detected in 72% of cases (18 out of 25 patients). ROC curve analysis demonstrated that *SOX2* gene CNV at a cutoff point of  > 3 can detect NSCLC with a sensitivity and specificity of 72% and 96% respectively, AUC was 0.8.

### *HV2* gene copy number variation

As regards *HV2* gene CNV, decreased copy number was also found in 72% of cases. For *HV2* gene CNV at a cutoff point of < 2, AUC was 0.775, *P* = 0.001 with a sensitivity and specificity of 72% and 84%, respectively for detection of NSCLC.

**Fig. 2:**
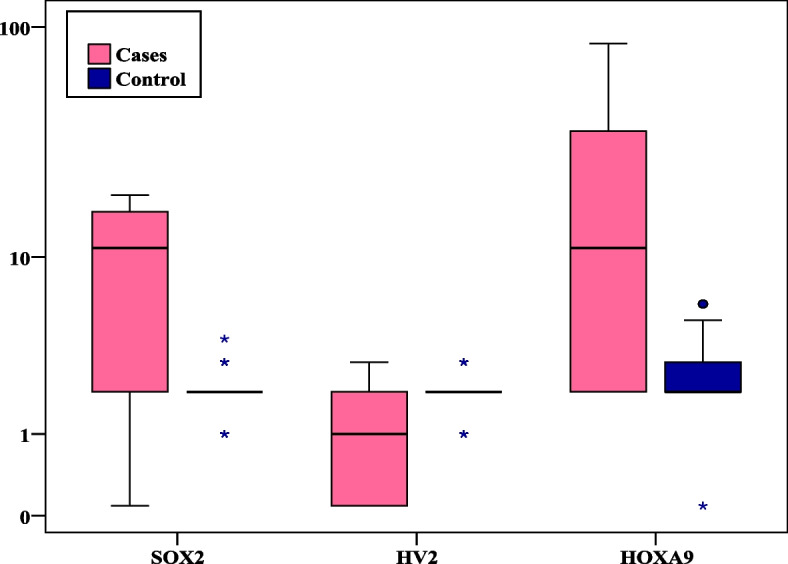
*SOX2 gene CNV*, *HV2* gene CNV and *HOXA9* gene promotor methylation in NSCLC cases and controls

Statistically significant difference was detected between the two studied groups as regards the three studied genes (Table 2), (Fig. 2).

**Table 2:** Comparison between the two studied groups according to *HOXA9* gene promotor methylation*, SOX2* gene and *HV2* gene copy number variation

	**NSCLC Cases** **(*n* = 25)**	**Controls** **(*n* = 25)**	**U**	***P***
*HOXA9* promotor methylation				
Min. – Max	2.0 – 85.0	0.0 – 6.0		
Median (IQR)	11.0 (2.0 – 36.0)	2.0 (2.0 – 3.0)	128.50^*^	< 0.001^*^
^a^ > 5%methylation	15	3		
≤ 5%methylation	10	22		
*SOX2* CNV				
Min. – Max	0.0 – 19.0	1.0 – 4.0		
Median (IQR)	11.0 (2.0 – 16.0)	2.0 (2.0 – 2.0)	125.0^*^	< 0.001^*^
^a^ > 3 copy number	18	1		
≤ 3 copy number	7	24		
*HV2* CNV				
Min. – Max	0.0 – 3.0	1.0 – 3.0		
Median (IQR)	1.0 (0.0 – 2.0)	2.0 (2.0 – 2.0)	140.50^*^	0.001^*^
^a^ < 2 copy number	18	4		
≥ 2 copy number	7	21		

*IQR* Inter quartile range, *SD* Standard deviation, *U* Mann Whitney test

^a^Cutoff values were calculated using ROC curve analysis

^*^Statistically significant at *p* ≤ 0.05

Out of the cases group, 8 patients had abnormalities in the three genes, while 11 patients had abnormalities in 2 genes (6 patients with *SOX2* and *HV2* CNV, 3 patients with *HV2* CVN and *HOXA9* hypermethylation and 2 patients with *SOX2* CVN and *HOXA9* hypermethylation), 5 patients had abnormalities in only one gene (2 patients with *SOX2* CVN, 2 patients with *HOXA9* hypermethylation and 1 patient with *HV2* CVN) whereas one patient didn't have any abnormal findings. In control group, 7 had single gene abnormality: 1 (*SOX2* CVN), 3 (*HV2* CVN), 3 (*HOXA9* hypermethylation).

### Associations between *SOX2 gene CNV*, *HV2* gene CNV and *HOXA9* gene promotor methylation and clinicopathological data

No statistically significant associations were found between *SOX2* gene *CNV*, *HV2* gene CNV and *HOXA9* gene promotor methylation and the following characteristics: age, sex, smoking status, pathological classification or clinical stage. No correlation was detected between the 3 markers (Table 3).

**Table 3:** Association between *HOXA9* gene promotor methylation, *SOX2 gene*, *HV2* gene copy number variation and clinicopathological data

	No	*HOXA*9% methylation	Test of Sig	*P*	*SOX2* CNV	Test of Sig	*P*	*HV2* CNV	Test of Sig	*P*
		Median (Min. – Max.)			Median (Min. – Max.)			Median (Min. – Max.)		
Age (years)		(30.0 – 75.0)	r_s_ = 0.389	0.055	(30.0 – 75.0)	r_s_ = -0.138	0.512	(30.0 – 75.0)	r_s_ = -0.025	0.907
Sex										
Male	23	13.0 (2.0 – 85.0)	U = 11.0	0.280	11.0 (0.0 – 19.0)	U = 12.50	0.327	1.0 (0.0 – 3.0)	U = 11.50	0.280
Female	2	3.50 (2.0 – 5.0)			14.50 (12.0 – 17.0)			2.0 (1.0 – 3.0)		
Smoking										
Non-smoker	2	3.50 (2.0 – 5.0)	U = 11.00	0.280	14.50 (12.0 – 17.0)	U = 12.50	0.327	2.0 (1.0 – 3.0)	U = 11.50	0.280
Smoker	23	13.0 (2.0 – 85.0)			11.0 (0.0 – 19.0)			1.0 (0.0 – 3.0)		
Pathology										
SCC	11	11.0 (2.0 – 85.0)	U = 73.00	0.851	5.0 (0.0 – 18.0)	U = 57.50	0.291	1.0 (0.0 – 3.0)	U = 74.0	0.893
Adenocarcinoma	14	10.0 (2.0 – 64.0)			12.0 (1.0 – 19.0)			1.0 (0.0 – 3.0)		
Staging										
I + II	5	33.0 (2.0 – 41.0)	U = 37.50	0.408	5.0 (0.0 – 12.0)	U = 27.00	0.129	0.0 (0.0 – 3.0)	U = 37.50	0.408
III + IV	20	11.0 (2.0 – 85.0)			12.50 (0.0 – 19.0)			1.0 (0.0 – 3.0)		

*U* Mann Whitney test, *p* p value for comparing between different categories, *r*_*s*_ Spearman coefficient

### Combined Receiver Operating Characteristic (ROC) curve analysis for the 3 molecular markers

**Fig. 3:**
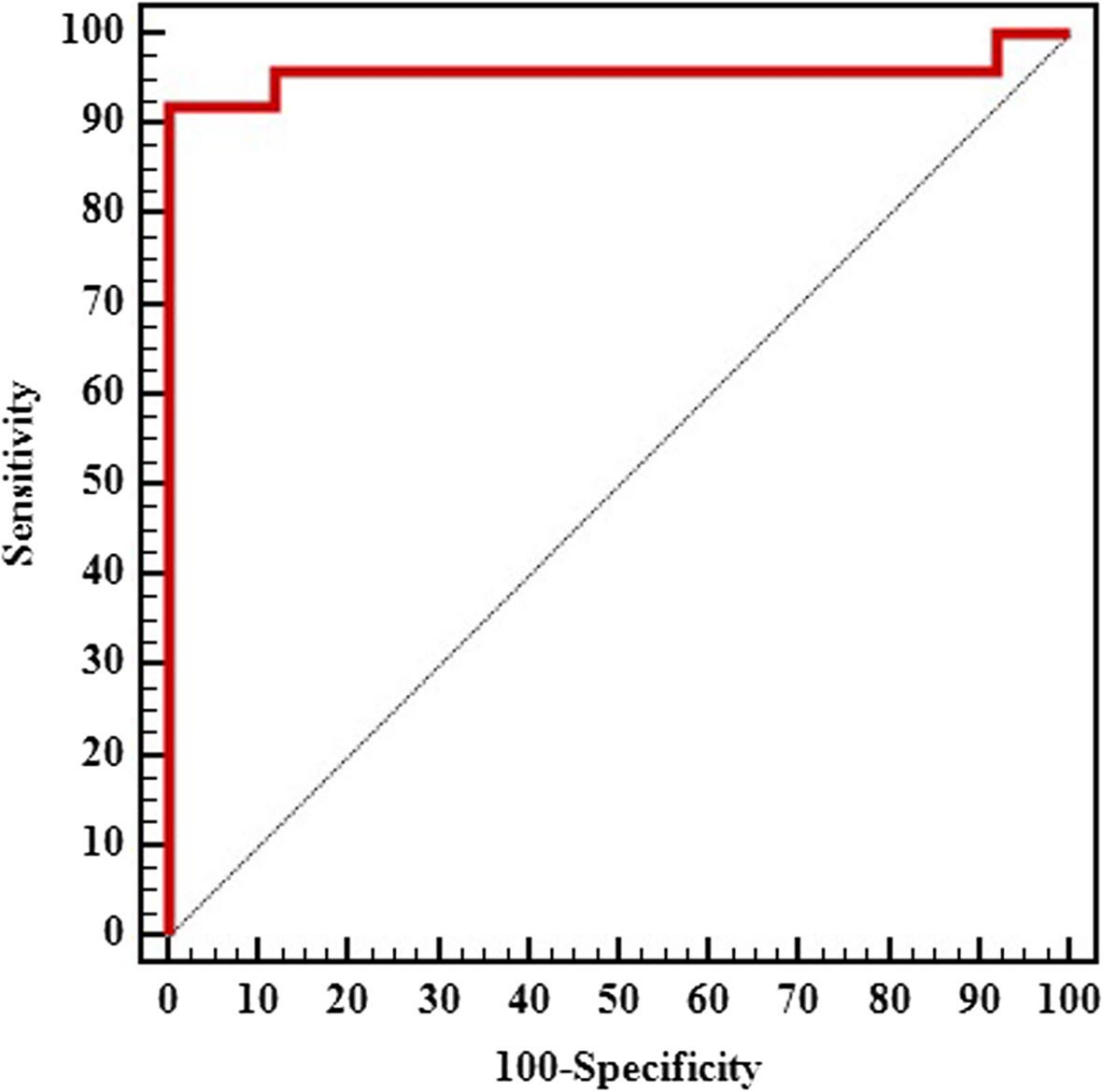
ROC curve for combined *SOX2 gene CNV*, *HV2* gene CNV and *HOXA9* gene promotor methylation to discriminate patients with lung cancer (*n* = 25) from controls (*n* = 25)

The combined analysis of the three genes revealed that AUC was 95.8%, p < 0.001. This indicates that the combined analysis of *SOX2* and *HV2* CNV and % *HOXA9* methylation could have a discrimination power of 95.8% between patients with NSCLC from control. Sensitivity and specificity were 88% and 100%, respectively (Fig. 3).

## Discussion

Unfortunately, the incidence of lung cancer stays high worldwide. Early detection of lung cancer in an early stage is still a clinical problem that needs to be solved urgently. Unlike the traditional tissue biopsy, cfDNA is noninvasive and real-time, cfDNA released from neoplastic cells can be detected at an early stage, making early diagnosis of cancer possible [20]. Genetic and epigenetic markers in cfDNA such as gene mutations, CNV and DNA methylation could be used as candidate biomarkers for diagnosis and risk stratification of lung cancer [21].

In this study, the diagnostic performance of *HOXA9* gene promoter methylation, *SOX2* gene and *HV2* gene CNV were evaluated for detection of lung cancer. Although we found statistically significant difference in the three biomarkers between patients and controls, individual gene sensitivity was somewhat limited. Upon performing ROC curve analysis, we found that *HOXA9* gene promoter methylation can detect NSCLC with 60% sensitivity and 88% specificity. *SOX2* gene CNV can detect NSCLC with 72% sensitivity and 96% specificity, while, *HV2* gene CNV that can discriminate NSCLC patients from controls with 72% sensitivity and 84% specificity. However, by combining the 3 genes in a panel, the diagnostic sensitivity and specificity were greatly improved to 88% and 100%, respectively. No associations were found between the three biomarkers and any clinicopathological data.

Several studies have evaluated the diagnostic performance of *HOXA9* gene promoter methylation as part of a diagnostic panel for lung cancer. Wrangle J, et al. examined the methylation status of *CDO1*, *HOXA9*, and *TAC1* genes in three cohorts of NSCLC tissues. They found that the methylation of the 3 gene panel is highly sensitive for the early diagnosis of NSCLC (83 to 99%) and the specificity of This three-gene panel is 100% [22]. Similar results were found by Yang Z, et al. who examined methylation of eight genes including *HOXA9* in plasma cfDNA by methylation specific PCR in lung cancer patients and inflammatory pseudo tumor cases. The overall sensitivity was 74% but it was lower for separate genes and the overall specificity was 91% [6]. Another study evaluated the promoter methylation of several genes in urine and plasma from pathologically confirmed NSCLC patients and healthy subjects. The sensitivity and specificity for *HOXA9* methylation in plasma cfDNA were 58% and 80% respectively independent of age, sex and smoking status. Univariate and multivariate regression analysis for *HOXA9* in plasma showed its association with risk of NSCLC [23]. Moreover, the methylation level of 4 genes including *HOXA9* were assessed using quantitative MSP in lung cancer tissue samples, plasma samples from primarily lung cancer patients and benign lung lesions. They found that *HOXA9* methylation is significantly higher in lung cancer cases but, in contrast to our findings *HOXA9* methylation levels were higher in squamous cell carcinoma in comparison with adenocarcinoma in lung cancer tissue samples. They proposed the use of cfDNA for lung cancer subtyping [24].

As regards *SOX2* gene, *SOX2* gene is the most important gene among all the SOX family of genes, due to its ability to reprogram somatic cells into induced pluripotent stem cells [16]. Kutilin D, et al. found a significant increase in *SOX2* CNV in 50% of lung tissue samples and it was 3.6 times higher in plasma samples compared to controls [25]. Another study demonstrated increase in the CNV of *SOX2* gene by fluorescence in situ hybridization (FISH) using tissues resected from NSCLC patients. They proposed that increased *SOX2* copy number may be an independent favorable prognostic factor regardless of histological classification [26]. Moreover, the copy number of *SOX2* and *TP53* were analyzed by quantitative real time PCR in tumor tissues and adjacent non-tumor tissues. Increased copy number was found in 34% of tumor tissues compared to non-tumor tissues [27]. Ying J, et al. also detected high expression of *SOX2* gene in NSCLC tissue samples compared to para-carcinoma tissues. They also found that the high expression was independent of age, gender, smoking status or TNM stage, but in contrast to our findings they found its association with histopathological type. The high *SOX2* expression was found in 50% of squamous cell carcinoma and 20.3% in adenocarcinoma [28].

A considerable number of research has been done exploring *HV2* gene CNV in lung cancer. Mitochondrial copy number variations and mitochondrial DNA mutations are reported to initiate a sequence of events that contribute to defect in oxidative phosphorylation, continuous increase in reactive oxygen species production and eventually cancer development [29]. Kutilin D, et al. found a decreased *HV2* gene CNV in 28% of lung cancer tissue samples. *HV2* gene CNV was 13.9 times lower in plasma samples and the number was even lower in metastatic patients by 1.4 times and 12.5 times in tissue and plasma samples respectively. They suggested the use of *SOX2* and *HV2* genes CNV as early non-invasive molecular diagnostic biomarkers [25]. In another study, the CNV of 10 genes including *HV2* gene were examined in plasma samples from histologically confirmed lung adenocarcinoma patients by quantitative PCR. They found a 16-fold decrease in *HV2* copy number in patients’ samples compared to healthy control and supported its use as an early diagnostic marker [30]. However, in contrast to our findings, they found significant decrease in the copy number in metastatic compared to non-metastatic patients. They presumed the use of these markers as markers to predict the risk of metastasis [18].

## Conclusion

Based on previous findings, we concluded that HOXA9 gene methylation and the CNV of SOX2 and HV2 genes in cfDNA could be used in a single panel for non-invasive diagnosis of NSCLC with high sensitivity and specificity. Future studies with a larger sample size are required to confirm the diagnostic utility of these three biomarkers in NSCLC.

## Data Availability

The data supporting the conclusions are included within the article.

## Abbreviations

BCL2L1: B-cell lymphoma 2 like1
cfDNA: Cell free DNA
CNV: Copy number variation
CT: Computed tomography
ctDNA: Circulating tumor DNA
*EGFR*: Epidermal growth factor receptor gene
EMT: Epithelial mesenchymal transition
FISH: Fluorescence in situ hybridization
*HOXA9*: Homeobox A9 gene
*HV1*: Hypervariable region 1 gene
*HV2*: Hypervariable region 2 gene
IQR: Interquartile range
MSP: Methylation sensitive PCR
mtDNA: Mitochondrial DNA
NSCLC: Non-small cell lung cancer
ROC: Receiver operating characteristic
RQ: Relative quantitation
SCC: Squamous cell carcinoma
SCLC: Small cell lung cancer
*SOX2*: Sex determining region Y-box 2 gene
